# A Mayer-Rokitansky-Kuster-Hauser patient with leiomyoma and dysplasia of neovagina: a case report

**DOI:** 10.1186/s12905-020-01026-1

**Published:** 2020-07-28

**Authors:** Varpu Jokimaa, Johanna Virtanen, Harry Kujari, Seija Ala-Nissilä, Virpi Rantanen

**Affiliations:** 1 Department of Obstetrics and Gynecology, Turku University Hospital and University of Turku, PL 52, 20521 Turku, Finland; 2 Department of Radiology, Turku University Hospital and University of Turku, 20521 Turku, Finland; 3 Institute of Biomedicine, Research Center for Cancer, Infections and Immunity, University of Turku, 20014 Turku, Finland; 4 Department of Pathology, Turku University Hospital, 20521 Turku, Finland

**Keywords:** MRKH, Leiomyoma, Dysplasia, Neovagina, Imaging, Case report

## Abstract

**Background:**

Most patients with congenital uterus and vaginal aplasia (i.e., Mayer–Rokitansky–Kuster–Hauser [MRKH] syndrome) have rudimentary pelvic uterine structures that contain smooth muscle. Although leiomyomas and dysplasia of vaginal mucosa are relatively common in the general population, they are rare in MRKH patients. Data on the vulnerability of neovaginas to HPV-associated dysplasia are limited.

**Case presentation:**

A rare case of an MRKH patient with two gynaecological conditions detected during long-term gynaecological follow-up is presented. At the age of 21, the patient was treated for HPV-associated neovaginal dysplasia. At the age of 47, a pelvic leiomyoma was detected with transvaginal ultrasound and confirmed with magnetic resonance imaging.

**Conclusion:**

A Pap smear or human papillomavirus testing is indicated in sexually active MRKH women. Uterine rudiments contain smooth muscle, which facilitates the development of oestrogen-dependent diseases, such as leiomyomas and adenomyosis. Although magnetic resonance imaging is recommended in cases of a pelvic mass, easily attainable and cost-efficient transvaginal ultrasound offers high diagnostic accuracy in patients with a surgically created neovagina and is suitable for the patients’ follow-up. Guidelines for the gynaecological follow-up of MRKH patients are warranted.

## Background

Aplasia of the uterus and vagina is a rare congenital malformation caused by the defective fusion of the Mullerian ducts during early foetal development. In Finland, the incidence of this malformation is one in every 5000 new-born girls [1]. Most of these patients have normal secondary sexual characteristics and a female karyotype, with skeletal and renal malformations in some cases. In most cases, rudimentary uterine structures are present in the pelvis (Mayer–Rokitansky–Kuster–Hauser [MRKH] syndrome), and total Mullerian aplasia is uncommon [1].

The follow-up practices used for MRKH patients are inconsistent. Additionally, how actively MRKH women participate in gynaecological screening programs and how common gynaecological morbidities are in this patient group are unknown. This case report presents a rare case of an MRKH patient with two gynaecological conditions: dysplasia of the neovagina and a benign pelvic leiomyoma.

## Case presentation

The patient was examined for primary amenorrhea at the age of 16, and diagnosis of MRKH syndrome was confirmed by laparoscopy, which showed bilateral normal ovaries and fallopian tubes connected to a fibrous band above the rectum. The patient’s ovarian histology was normal. Urinary system malformations were excluded, and no skeletal malformations were observed. When the patient was 17 years old, a neovagina was created without complications using Davidov’s technique. Post-operatively, the neovagina was 6 cm long, and the vaginal opening was the width of two fingers. Coital activity began within 2 months of the operation. During follow-up, the neovagina appeared functionally and structurally normal.

**Fig. 1:**
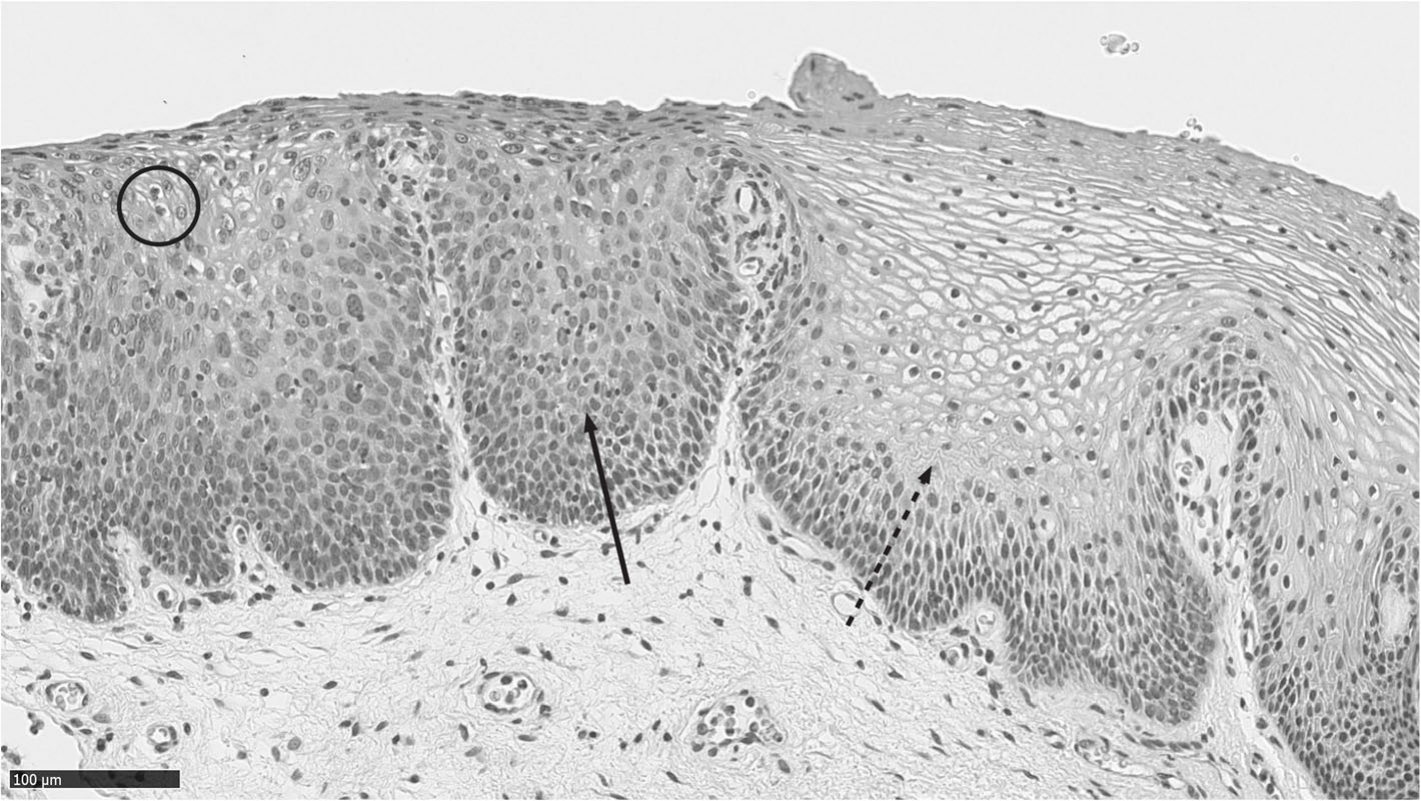
In the microphotograph, the squamous epithelium is slightly thickened and occupied by HSIL level dysplastic epithelium on the left (solid arrow). Koilocytes can be seen on the surface (circle). The epithelium is essentially normal on the right side of the photograph (dotted arrow)

At the age of 21 years, a colposcopy following an abnormal Pap smear (Papanicolaou class III) revealed mostly normal-looking mucosa as well as small, bilateral areas of pale, thick papillary mucosa. A biopsy revealed a high-grade intraepithelial lesion (VAIN 2; Fig. 1), and human papillomavirus (HPV) testing was positive for HPV type 16. The dysplasia and a subsequent recurrence were successfully treated by laser vaporization. The patient’s Pap smears remained normal until the age of 47 years, when virus testing was positive for high-risk HPV types other than 16 or 18. Biopsies revealed an HPV infection in the neovaginal epithelium and a low-grade intraepithelial lesion of the vulva, both suitable for conservative follow-up.

**Fig. 2:**
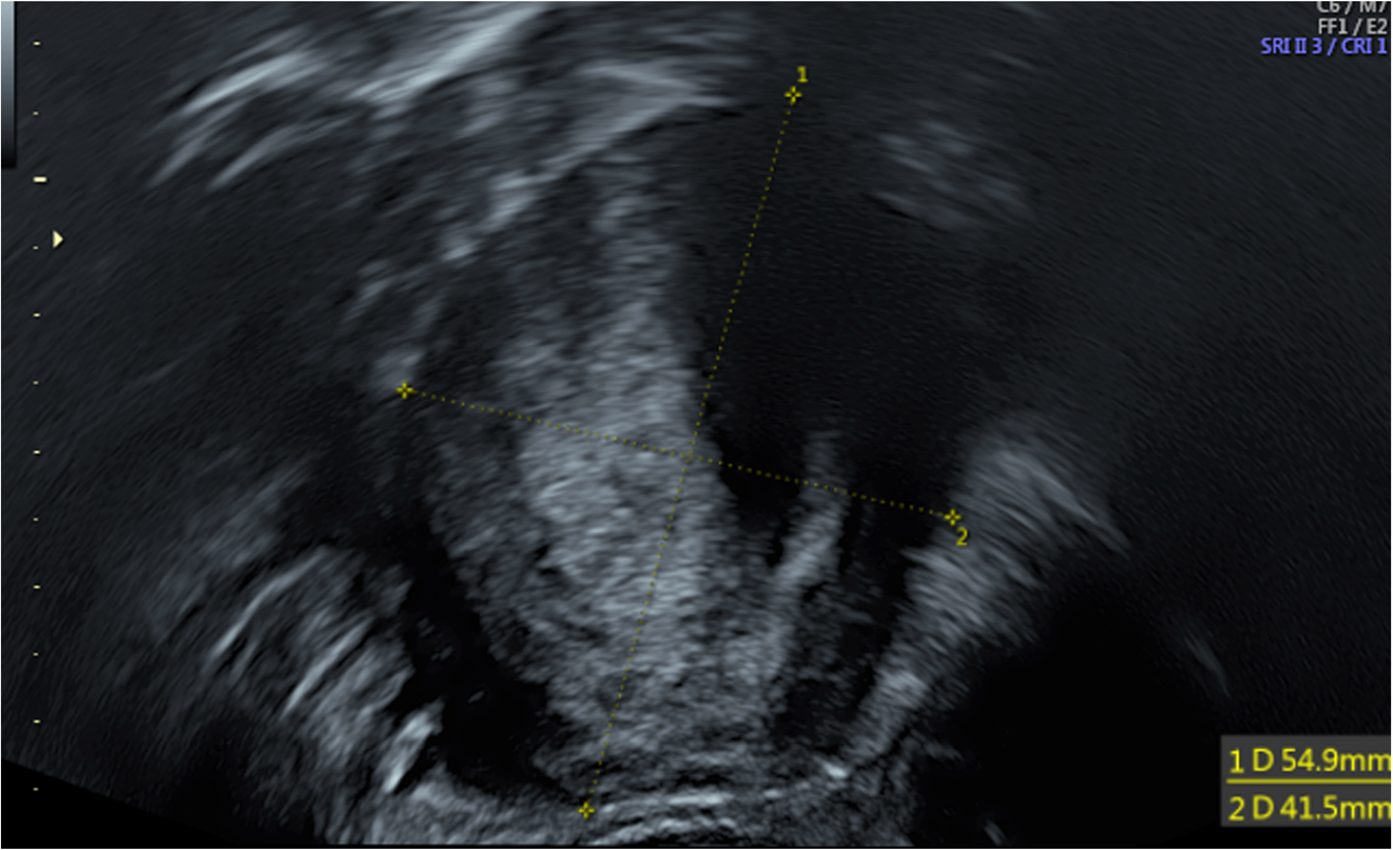
Transvaginal ultrasound revealed heterogenic pelvic mass and acoustic shadows typical for leiomyoma. (Voluson S10, GE Health, Chicago, Illinois, USA)

Also at the age of 47 years, a mobile pelvic mass was detected in a routine follow-up visit. A transvaginal ultrasound examination revealed a right-sided solid tumour (diameter: 55 mm; Fig. 2). A small homogeneous area suggestive of uterine rudiments was visible lateral to the tumour. Moderately variable tumour echogenicity and acoustic shadows indicated a leiomyoma. However, as the right ovary was not visible in the examination, the diagnosis remained uncertain.

**Fig. 3:**
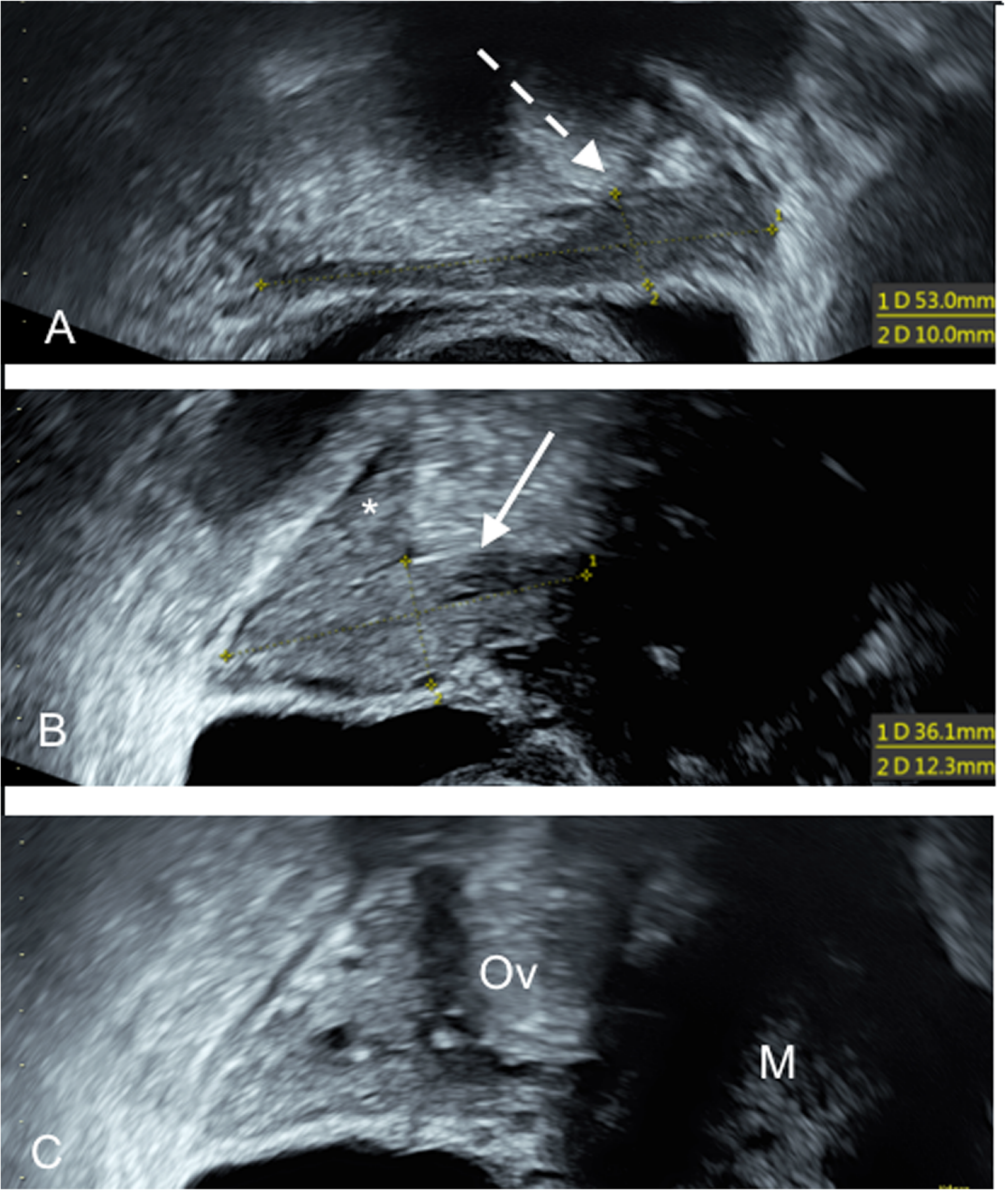
**a** A tear-shaped uterine remnant (dotted arrow) on the left side visualized by transvaginal ultrasound. **b** The right-sided uterine remnant (arrow) and the lower pole of ovary (asterixis) immediately above the uterine remnant. **c** Right ovary (Ov) and leiomyoma (M). (Voluson S10, GE Health, Chicago, Illinois, USA)

Ovarian tumour markers (CA125: 9 kU/L, HE4: 35 pmol/L and CEA: 2.9 μg/L) were normal. At a follow-up 4 weeks later, the tumour measured 60 × 52 mm, and Doppler ultrasound showed sparse blood flow in the tumour. Uterine rudiments and ovaries with small antral follicles were identified bilaterally (Fig. 3). The bladder and ureters were normal, and peristalsis and the jet from the right ureter were normal. The rectovaginal septum was even and of normal thickness.

**Fig. 4:**
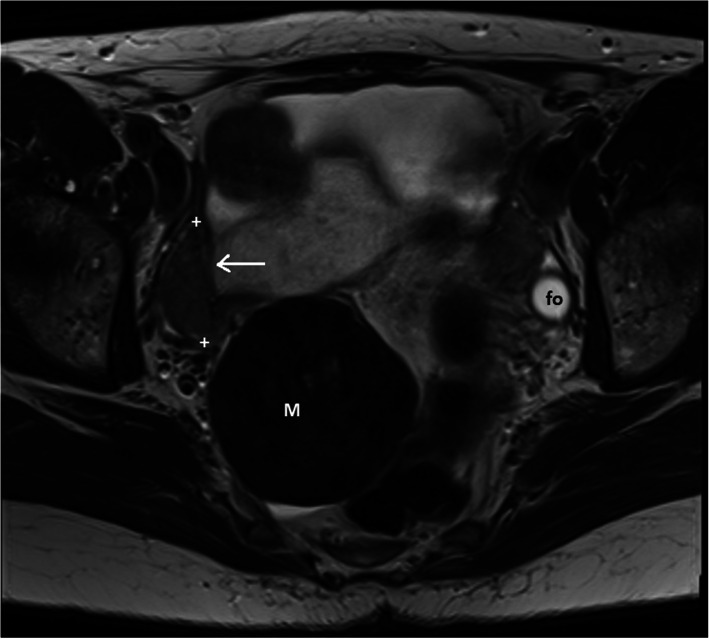
T2 weighted high-resolution MRI in axial plane showing the typical radiological features of a leiomyoma (M) and the uterine remnant at the right side of the patient (arrow). Follicle in the left ovary (fo). MRI was conducted with a 3 T MRI scanner with integrated body coil, spine and body matrix receiving surface coils. Siemens (Skyra Fit, Siemens Medical Solutions, Erlangen, Germany)

Magnetic resonance imaging (MRI) confirmed the ultrasonographic findings, showing a well-circumscribed 60-mm pelvic tumour, normal ovaries and bilateral uterine rudiments connected by a transversal tissue band crossing the pelvis (Fig. 4). The low signal intensity of the tumour was typical of a benign leiomyoma. As the leiomyoma was asymptomatic and showed no signs of malignancy, conservative follow-up was recommended. The leiomyoma remained unchanged at follow-up after 18 months.

## Discussion and conclusion

The literature on the long-term gynaecological health of MRKH patients is limited. This case report presented an MRKH patient who, during a follow-up of 30 years, developed two gynaecological conditions: dysplasia of the neovagina and a benign pelvic leiomyoma. The presented case highlights the need for gynaecological follow-up of MRKH patients, including pelvic imaging, Pap smears and testing for HPV infections.

Neovaginas can be created through dilatation of the vaginal dimple, using peritoneal distension (Davidov’s technique) and fasciocutaneous flaps, or they can be crafted from the intestines or skin. Histological changes may occur because the grafted tissue is subject to new stressors, such as exposure to semen or micro-injuries during intercourse exposing the basal membrane, the primary binding site for HPV. The vulnerability of particular tissues to HPV infections and premalignant changes remains unclear, as only case studies of dysplasia and neovaginal cancers have been published [2–5]. It is suggested that squamous cell cancers occur in skin-graft neovaginas, whereas adenocarcinomas occur in intestinal neovaginas [2, 4], but most reports have not investigated MRKH patients operated with Davidov’s method.

Additionally, the literature on the prevalence of HPV positivity in neovaginas is limited [3, 5, 6], but the rate of occurrence seems similar to that in the general population [6]. The present case demonstrated that serial HPV infections may also occur in MRKH women. Therefore, HPV-associated lesions should be considered in sexually active patients with a neovagina who report bleeding or discharge. Participation in cervical cancer screening programs is recommended, although no guidelines exist for the follow-up of these patients. Additionally, vaccination of MRKH patients against HPV is recommended [3, 6]. Neovaginal dysplasia can be treated with laser vaporization, as in the present case, or excision.

Uterine rudiments contain smooth muscle, which facilitates the development of oestrogen-dependent diseases, such as leiomyomas and adenomyosis [7]. Leiomyomas are relatively common in the general population but are rarely detected in MRKH patients; only a few case reports have presented leiomyomas originating from Mullerian remnants [7–9]. The differential diagnosis of pelvic masses in MRKH patients includes ovarian tumours, gastrointestinal stromal intestinal tumours and leiomyomas arising from uterine rudiments or the urinary bladder [9]. Malignant transformation of leiomyomas in MRKH patients has not been reported.

In the literature, the diagnoses of leiomyomas in MRKH patients were based on transabdominal ultrasound or MRI and were confirmed at the time of surgery in most cases. Uterine sarcomas cannot be definitively excluded based on clinical symptoms or growth pattern but MRI can distinguish between usual leiomyomas and extremely rare leiomyosarcomas in most cases [10]. In the present case, the well-defined margins, regular oval shape and low T2 signal intensity were typical of a benign leiomyoma [10]. According to the updated guidelines, only symptomatic leiomyomas require treatment [11], and this principle is especially true for women approaching menopause, like the patient of this case report, as leiomyomas typically diminish after menopause.

The diagnosis of uterus aplasia was based on laparoscopy until the advent of MRI, but MRI is currently the diagnostic gold standard for severe uterine malformations. Indeed, laparoscopy is seldom needed to confirm the diagnosis, as MRI can detect even minor uterine structures [12–14]. In the present case, high-resolution transvaginal ultrasound provided high diagnostic accuracy, and MRI confirmed the ultrasonographic findings. Both imaging methods visualized the bilateral uterine remnants, leiomyoma and ovaries. Therefore, the less costly and more easily attainable ultrasound could be used in the follow-up of the patient.

A sufficient vaginal length is needed to ensure the diagnostic accuracy of a transvaginal ultrasound examination. Achieving adequate resolution may be difficult in cases in which the rudimentary uterine structures are located high in the abdominal cavity. Visualization of the pelvic structures may also be poor if the neovagina is not structurally or functionally optimal. In the present case, Davidov’s vaginoplasty and early initiation of coital activity resulted in a functionally and anatomically excellent outcome.

The strength of this case report is the long follow-up of the patient, which lasted over 30 years. Detailed documentation of the diagnostic measures and treatments were available from the patient’s diagnosis of MRKH at the age of 16. The presented case showed that common gynaecological disorders may also develop in patients with congenital aplasia of uterus and vagina. However, no conclusions on the prevalence of these disorders in such patients can be made based on case reports. Systematic multicentre studies are needed to create guidelines for the follow-up of MRKH patients.

In conclusion, common gynaecological disorders may occur in MRKH patients; therefore, these patients should be followed up similarly to other women. HPV-associated dysplasia may develop in a neovagina created from peritoneum or other tissues, and therefore, Pap smears or HPV testing is indicated in sexually active MRKH women. Additionally, uterine rudiments contain smooth muscle, which facilitates the development of leiomyomas. MRI is recommended in cases of a pelvic mass; however, high diagnostic accuracy is achieved using transvaginal ultrasound once the neovagina is of normal length, which enables the use of transvaginal ultrasound for follow-up.

## Data Availability

Not applicable.

## Abbreviations

CA125: Cancer antigen 125
CEA: Carcinoembryogenic antigen
HE4: Human epididymis protein 4
HPV: Human papilloma virus
MRI: Magnetic resonance imaging
MRKH: Mayer–Rokitansky–Kuster–Hauser syndrome
